# Socio-demographic patterning of the individual-level double burden of malnutrition in a rural population in South India: a cross-sectional study

**DOI:** 10.1186/s12889-020-08679-5

**Published:** 2020-05-13

**Authors:** Matthew Little, Sally Humphries, Warren Dodd, Kirit Patel, Cate Dewey

**Affiliations:** 1 Department of Population Medicine, University of Guelph, Guelph, ON Canada; 2 School of Public Health and Social Policy, University of Victoria, Victoria, BC Canada; 3 Department of Sociology and Anthropology, University of Guelph, Guelph, ON Canada; 4 School of Public Health and Health Systems, University of Waterloo, Waterloo, ON Canada; 5 Department of International Development Studies, Menno Simons College, University of Winnipeg, Winnipeg, MB Canada

**Keywords:** India, Rural, Malnutrition, Anemia, Overweight, Obesity, diabetes, Undernutrition, Over-nutrition, Double burden

## Abstract

**Background:**

The double burden of malnutrition is the co-occurrence of undernutrition (e.g. underweight, stunting, and micronutrient deficiencies) and over-nutrition (e.g. obesity, type 2 diabetes, and cardiovascular disease) at the population, household, or individual level. The objectives of this study were to determine the extent and determinants of individual-level co-morbid anemia and overweight and co-morbid anemia and diabetes in a population in rural Tamil Nadu, South India.

**Methods:**

We undertook a cross-sectional study of adults (*n* = 753) in a rural region of Tamil Nadu, South India. A survey assessed socio-demographic factors, physical activity levels, and dietary intake. Clinical measurements included body-mass index, an oral glucose tolerance test, and blood hemoglobin assessments. Multivariable logistic regression analyses were used to determine associations between risk factors and two co-morbid double burden pairings: (1) anemia and overweight, and (2) anemia and diabetes.

**Results:**

Prevalence of co-morbid anemia and overweight was 23.1% among women and 13.1% among men. Prevalence of co-morbid anemia and diabetes was 6.2% among women and 6.3% among men. The following variables were associated with co-morbid anemia and overweight in multivariable models [odds ratio (95% confidence interval)]: female sex [2.3 (1.4, 3.85)], high caste [3.2 (1.34, 7.49)], wealth index [1.1 (1.00, 1.12)], rurality (0.7 [0.56, 0.85]), tobacco consumption [0.6 (0.32, 0.96)], livestock ownership [0.5 (0.29, 0.89)], and energy-adjusted meat intake [1.8 (0.61, 0.94)]. The following variables were associated with co-morbid anemia and diabetes in multivariable models: age [1.1 (1.05, 1.11)], rurality [0.8 (0.57, 0.98)], and family history of diabetes [4.9 (1.86, 12.70).

**Conclusion:**

This study determined the prevalence and factors associated with individual-level double burden of malnutrition. Women in rural regions of India may be particularly vulnerable to individual-level double burden of malnutrition and should be a target population for any nutrition interventions to address simultaneous over- and undernutrition.

## Background

Low- and middle-income countries (LMICs) around the globe are undergoing a nutrition transition, characterized by shifting dietary and physical activity patterns [1–3]. In India, dietary changes include increased intakes of vegetable oils, refined grains, and processed foods, as well as reduced consumption of legumes and coarse cereals [4, 5]. Levels of inactivity are also rising as manual labour is replaced by sedentary work, and leisure activities remain relatively inaccessible and unpopular [4]. The combined result of such dietary and lifestyle changes has been a population-level increase in over-nutrition leading to obesity and associated diseases. Indeed, prevalence of obesity and overweight increased in excess of 125% from 2003 to 2015 and is currently 30–40% in some urban populations and 15–30% in some rural populations [6, 7]. Consequently, obesity-related cardio-metabolic diseases are becoming severe public health concerns in India; for example, prevalence of type 2 diabetes is 10–18% in urban and 5–13% in rural populations [8–10], while mortality due to ischemic heart disease and stroke account for 21% of all deaths [11].

Meanwhile, despite rapid economic development and associated dietary and lifestyle changes, many regions of India continue to experience poverty, food insecurity, and poor access to health services, which contribute to persistent problems of undernutrition and related deficiencies. India has the highest number of severely undernourished people in the world (190 million), representing 15% of its entire population [12]. Approximately 23% of women and 20% of men (age 15–49) are underweight [7]. Additionally, micronutrient deficiencies and associated disorders affect a large portion of the population, particularly in rural regions, where 70% of Indians reside [7, 13]. In particular, iron-deficiency anemia affects 53.1% of women of childbearing age and is a widely used marker of undernutrition [7].

The combined burdens of overweight and obesity-related diseases, in addition to undernutrition and micronutrient deficiencies, is called the ‘double burden’ of malnutrition. This double burden is common in LMICs undergoing the nutrition transition and has been reported in Latin America [14–16], South Asia [17], Southeast Asia [2, 18], Eastern Europe [2], and Africa [19, 20]. In India, the double burden of malnutrition is well-established at the national level [7]; however, the co-occurrence of over-nutrition and undernutrition also exists at the household and individual levels [21, 22]. Individual-level double burden may occur when a person experiences co-morbid indicators of over-nutrition (e.g. obesity, type 2 diabetes, and cardiovascular disease) and undernutrition (e.g. underweight, stunting, and micronutrient deficiencies). Yet, the individual-level double burden of malnutrition has received limited attention by researchers, especially in rural regions of India where poverty, food insecurity, and poor access to healthcare services are pervasive despite rapid changes in diets, lifestyles, and livelihoods [23]. Against this backdrop, this cross-sectional study had two objectives. First, we evaluated the extent of the individual-level double burden of malnutrition in a rural region of northwestern Tamil Nadu, India by assessing two co-morbidities: (1) anemia and overweight; and (2) anemia and diabetes. Second, we determined associations between these co-morbidities and several socio-economic, environmental, dietary, and lifestyle factors. Overall, we aim to contribute to research on the severity and determinants of the individual-level double burden of malnutrition in rural South India.

## Methods

### Study design and sample

**Fig. 1:**
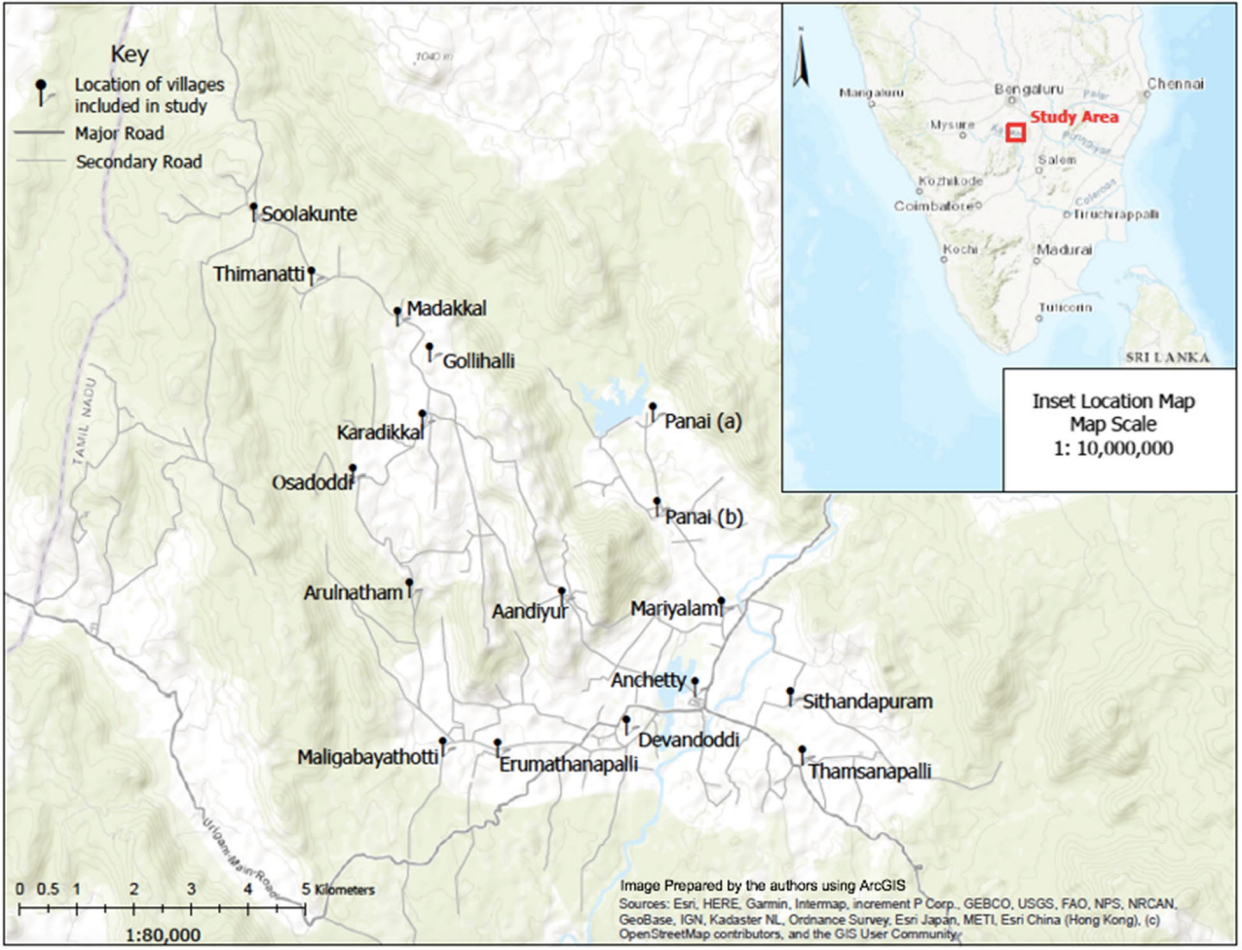
Location of study area and villages included in the study

We conducted a cross-sectional study in 18 villages in Anchetty and Madakkal *panchayats* (townships) in the Krishnagiri district, in northwestern Tamil Nadu (Fig. 1). Both Tamil and Kannada are spoken in the study region due to its proximity to Karnataka. The Krishnagiri district is ranked as one of the poorest districts in Tamil Nadu, with high illiteracy, low gross district domestic product, and a low gender development index compared to other districts in Tamil Nadu [24]. Within the study site, rates of illiteracy and poverty are higher than the district averages; 48.3% of adults are illiterate and 36% live below the poverty line [25].

Details of the study design are described elsewhere [26]. Briefly, recruitment of individuals occurred through a randomized two-stage method, in which we approached a random sample of 8% of households in the sampling frame, then employed World Health Organization (WHO) Kish method to select a single household member (> 19 years of age) for the study [27]. Pregnant women were excluded. During follow-up appointments with participants, we collected data on descriptive characteristics using a semi-quantitative survey, dietary intake using a validated food frequency questionnaire (FFQ) [28], and physical activity habits using the WHO’s global physical activity questionnaire (GPAQ) [29].

### Clinical measurements, definitions, and variables

Weight, height, waist circumference, and hip circumference were measured for each participant using standardized techniques and body mass index (BMI, kg/m^2^) was calculated [30]. Blood pressure was recorded as the average of two readings taken on the right arm in the sitting position with a portable OMRON BP-760 electronic blood pressure monitor (Omron Healthcare, Hoofddorp, Netherlands). Glucose tolerance was determined using an oral glucose tolerance test. After an 8-h minimum overnight fast, we measured fasting capillary blood glucose (CBG) with a One Touch Ultra glucometer (Johnson & Johnson, Milpitas, CA, USA). Oral glucose (75 g anhydrous) was administered and consumed within 5 min. Two hours later, we measured post-load CBG [31]. Blood hemoglobin (Hb) concentration was assessed using capillary blood samples and a HemoCue® HB201+ analyzer (Hemocue AB, Angelholm, SE).

Participants were categorized into BMI classes using the cut-offs for Asian populations: underweight (< 18.5 kg/m^2^); normal (≥18.5 kg/m^2^ and < 23 kg/m^2^); overweight (≥23 kg/m^2^ and < 25 kg/m^2^); obesity class I (≥ 25 kg/m^2^ and < 30 kg/m^2^); and obesity class II (≥35 kg/m^2^) [32]. Abdominal obesity was defined as waist circumference ≥ 90 cm for men and ≥ 80 cm for women [33]. Consistent with previous studies [21, 34], participants were classified as stunted if their height was <− 2 Z-scores below the sex-specific reference population at 18 years of age, calculated as < 163.6 cm for men and < 151.8 cm for women [35]. High blood pressure was defined as mean systolic blood pressure ≥ 140 mmHg and/or mean diastolic blood pressure ≥ 90 mmHg and/or self-reported treatment with blood pressure medication [36]. As per WHO criteria, diabetes was defined as individuals with proof of previous diagnosis and/or fasting CBG ≥7 mmol/L (≥126 mg/dl) and/or a 2 h post prandial CBG value ≥12.2 mmol/L (≥220 mg/dL) [31]. Impaired glucose tolerance (IGT) was defined as a fasting CBG < 7 mmol/L and a 2-h post glucose CBG ≥8.9 mmol/L (≥160 mg/dL) but < 12.2 mmol/L (220 mg/dL) [31]. Impaired fasting glucose (IFG) was defined as a fasting CBG ≥6.1 mmol/L (≥110 mg/dL) and < 7 mmol/L (< 126 mg/dL) and a 2 h post-glucose CBG < 8.9 mmol/L (< 160 mg/dL). Pre-diabetes was defined as the existence of IGT or IFG or co-occurrence of both. Mild anemia was defined as blood Hb concentration 110–129 g/L for men and 110–119 g/L for women. Moderate and severe anemia were defined as blood Hb concentration 80–109 g/L and < 80 g/L respectively for both men and women.

Socioeconomic status (SES) was determined with an asset-based wealth index (hereafter referred to as wealth index) using a subset of 13 of 29 questions taken from the Standard of Living Index developed by the International Institute of Population Sciences (IIPS) for use in their National Family and Health Surveys (NFHS) [37]. Attributes and possessions were weighted for a maximum score of 41 using weights developed by the IIPS [38]. Higher values were therefore indicative of a greater household asset base. Caste was categorized as low caste (comprised of scheduled castes and scheduled tribes), middle caste (comprised of other backward castes and most backward castes), high caste (Brahmin caste), or not applicable (in the case of non-Hindu religion).

### Data analysis

Food frequency questionnaire data were processed with EpiNu® (Madras Diabetes Research Foundation, Chennai, TN, IN), which provided information on caloric consumption (kilocalories per day) and average daily macro- and micronutrient intake (grams per day). Nutrient intake variables were scaled to grams per 1000 kcal to account for differences in energy intake between participants. Physical activity scores were calculated using WHO’s GPAQ Analysis Guide, which provided a total measure of metabolic equivalent minutes per week [29]. Values were scaled to hours per day of moderate physical activity. Sedentary time was defined as hours spent sitting per day and television time was defined as hours spent watching television per day. We calculated a rurality index value for each individual and standardized these values to a mean of zero and a SD of one, adapted from Weinert & Boik [39]. A higher rurality index value represented the product of a greater degree of remoteness and lower population density of home village.

Statistical analyses were completed in STATA Version 13.0 (StataCorp, College Station, TX, USA). Clinical and sociodemographic characteristics of the study population were tabulated and compared by sex using Student’s t-tests for means and Pearson chi-squared tests for proportions. We calculated sex-specific prevalence of indicators of over-nutrition (overweight, obese class I, obese class II, pre-diabetes, diabetes, high blood pressure, and abdominal obesity) and undernutrition (underweight, mild anemia, moderate anemia, severe anemia, and stunted). Prevalence values were age- and sex-standardized using state-level age and sex data from the 2011 national census [40]. We then calculated sex-specific prevalence of two double burden pairs (DBP); DBP1 was co-morbid anemia and overweight or obese and DBP2 was co-morbid anemia and type 2 diabetes. We created a four-level categorical variable for each DBP. For DBP1, these categories were: 0 = neither anemia nor overweight/obese (referent); 1 = anemia only; 2 = overweight/obese only; and 3 = co-morbid anemia and overweight/obese. For DBP2, these categories were: 0 = neither anemia nor diabetes (referent); 1 = anemia only; 2 = diabetes only; and 3 = co-morbid anemia and diabetes. We calculated means and proportions of a variety of demographic, wealth, physical activity, and dietary characteristics for each category level. Differences in means and proportions were assessed using one-way analysis of variance (ANOVA) and Pearson’s chi-squared tests, respectively. If these tests yielded a statistically significant test result (*p* < 0.05), we employed a Sidak pairwise comparison to determine which categories were different from each other.

Using the logit command in STATA, we conducted a backward stepwise model-building process to develop multinomial logistic regression models using the categories of each DBP as dependent variables. We therefore built a total of three models for each DBP using the same method. First, age- and sex-adjusted bivariate models were assessed to determine factors associated with the outcome at a liberal *p*-value of 0.2 with anemia, overweight, and co-morbid anemia and overweight (for DBP1 model) or anemia, diabetes, and co-morbid anemia and diabetes (for DBP2 model). Factors that were associated at the liberal p-value were then included in initial multivariable logistic regression models. We eliminated non-significant variables using a p-value cut-off of 0.05 from each model, assuming no confounding if coefficients of remaining variables changed by less than 20% after removal of the variable. Quadratic terms and interaction terms were assessed if there was biological or practical justification. Multicollinearity was assessed in all models but was not determined to be present. All models were adjusted for age and sex.

## Results

A total of 812 individuals were recruited for the study. Of these, 753 ultimately participated, including 341 men and 412 women. Response rate was 87.4% among men and 99.2% among women. In total, 752 (92.6%) completed a FFQ and 749 (92.2%) participated in the oral glucose tolerance test and submitted capillary blood samples for hemoglobin assessment. The mean age of participants was 47 (range 20–92). Over three-quarters (75.7%) of women were illiterate, compared to just over half (50.4%) of men. On average, men were more educated than women as measured by years of schooling.

Sex-specific unadjusted clinical and sociodemographic characteristics are presented in Table 1. Age- and sex-standardized prevalence of underweight, overweight, obesity class I, and obesity class II among the study population were 22.7, 14.9, 16.1, and 3.3% respectively. Age- and sex-standardized prevalence of IFG, IGT, and type 2 diabetes were 3.9, 5.6, and 10.8% respectively. Of those with type 2 diabetes, 56.4% were previously undiagnosed. Age- and sex-standardized prevalence of mild, moderate, and severe anemia were 19.9, 22.6, and 4.8% respectively. Anemia (mild, moderate, or severe) affected a greater proportion of women (57.2%) than men (35.2%).

**Table 1:** Sex-specific clinical and sociodemographic characteristics of a sample of adults (> 19 years) in rural South India

Characteristic	Men n	Mean (SD) or %	Women n	Mean (SD) or %	***p***-value
Age (y)					0.10
20–34	69	20.2	95	23.1	
35–49	115	33.6	147	35.7	
50–64	105	30.7	111	26.9	
65+	52	15.2	58	14.1	
Height (cm)	340	165 (7.0)	407	154 (6.6)	< 0.001
Stunting (men/women< 163.6/151.8 cm)	120	35.3	129	31.7	0.26
Body Mass Index (kg/m^2^) (BMI)	340	21.6 (3.91)	406	22.0 (4.49)	0.10
BMI Categories					
Underweight (< 18.5)	84	24.7	85	20.9	
Normal weight (≥18.5 & < 23 kg/m)	139	40.9	162	39.9	
Overweight (≥23 and < 25 kg/m^2^)	56	16.5	62	15.2	
Obese class I (≥25 and < 30 kg/m2	55	16.2	77	19.0	
Obese class II (≥30 kg/m2)	6	1.8	20	4.9	
Waist circumference (cm)	337	82 (11.3)	407	78 (12.5)	< 0.001
Abdominal obesity categories (waist circumference)					
Non-obese (men/women < 90/< 80 cm)	252	74.8	236	58.0	
Obese (men/women ≥90/≥80 cm)	85	25.2	171	42.0	
Hemoglobin (Hb) (g/dL)	336	13.4 (2.48)	407	11.5 (5.77)	< 0.001
Anemia (Hb concentrations)					
No (men/women ≥130/≥120 g/dL)	215	64.0	171	42.0	
Mild anemia (men/women 110–129/110–119 g/dL)	75	22.3	74	18.2	
Moderate anemia (men/women 80–109/70–109 g/dL)	37	11.0	148	36.4	
Severe anemia (men/women < 80/< 70 g/dL)	9	2.7	14	3.4	
High blood pressure (SBP ≥ 140 mmHg and/or DBP ≥ 90 mmHg and/or treatment with blood pressure medication)	105	31.1	117	28.8	0.51
Glucose tolerance and diabetes					
Impaired fasting glucose (fasting CBG 6.1–6.9 mmol/L)	8	2.4	17	4.2	0.17
Impaired glucose tolerance (fasting CBG < 7 mmol/L and a 2-h post glucose CBG ≥8.9 mmol/L but < 12.2 mmol/L)	17	5.0	24	5.9	0.60
Diabetes (proof of previous diagnosis and/or fasting CBG ≥7 mmol/L and/or 2-h post prandial CBG value ≥12.2 mmol/L)	47	13.8	48	11.7	0.40
Education					< 0.001
Illiterate	174	51.0	315	76.5	
Literate, less than primary school	94	27.6	48	11.7	
Primary school	59	17.3	46	11.2	
Secondary/post-secondary school	14	4.1	3	0.7	
Wealth Index					< 0.001
Low	108	31.7	200	48.5	
Middle	217	63.6	188	45.6	
High	16	4.7	24	5.8	
Tobacco use					< 0.001
Current users	173	51.3	279	68.9	
Not current users	164	48.7	126	31.1	

*p*-values are for differences in means or proportions of each characteristic between sexes using two-sided Student’s t-tests and Pearson chi-squared tests, respectively

Double burden pairings were prevalent among participants (Table 2). Overall prevalence of co-morbid anemia and overweight or obesity was 23.1% among women and 13.1% among men. Meanwhile, prevalence of co-morbid anemia and pre-diabetes was 6.2% among women and 2.4% among men, and co-morbid anemia and diabetes was 5.9% among women and 6.3% among men. Only 12.9% of participants (8.7% of women and 17.9% of men) did not have any indicator of either over- or undernutrition (diabetes, overweight, obesity, abdominal obesity, hypertension, stunted, anemia, or underweight), indicating a small proportion of the population were adequately nourished and cardio-metabolically healthy.

**Table 2:** Double burden of malnutrition characterization in a sample of adults from rural South India

Double burden characterization	Men n	%	Women n	%	***p***-value
Anemia and overweight or obese	44	13.1	94	23.1	< 0.001
Anemia and pre-diabetes	8	2.4	25	6.2	0.01
Anemia and diabetes	21	6.3	24	5.9	0.84
Anemia and hypertension	40	12.0	67	16.6	0.07
Stunted and overweight	43	12.6	54	13.3	0.79
Stunted and pre-diabetes	7	2.1	15	3.7	0.19
Stunted and diabetes	19	5.6	16	3.9	0.29
Stunted and hypertension	32	9.5	40	9.9	0.85

*p*-values are for differences in proportions between sexes using Pearson chi-squared test

Descriptive characteristics of the study population by diagnostic category of DBP1 and DBP2 are displayed in Tables 3 and 4, respectively. A significant difference in means or proportions between categories of DBP1 was seen for several attributes, including age, sex, rurality, wealth index, physical activity habits, and dietary intake. Additionally, a significant difference in means or proportions between categories of DBP2 was seen for several characteristics, including age, sex, rurality, family history, religion, wealth index, physical activity, and dietary intake.

**Table 3:** Clinical, sociodemographic, and dietary characteristics by double burden diagnostic category in a sample of adults from rural South India

Characteristic	Category 1: Neither anemia nor overweight (***n*** = 250)	Category 2: Anemia only (***n*** = 218)	Category 3: Overweight or obese only (***n*** = 145)	Category 4: Co-morbid anemia and overweight or obese (***n*** = 138)	***p***-value for trend*
***Descriptive characteristics***					
Age	44.9 ± 15.0	51.5 ± 16.1	45.5 ± 11.7	46.2 ± 13.3	< 0.001^a,d,e^
Women (%)	41.7	65.1	49.0	68.1	< 0.001^a,c,d,f^
Hypertension (%)	22.4	24.1	41.5	40.1	< 0.001^a,b,d,e^
Rurality Index	−0.21 ± 1.31	−0.24 ± 1.14	−0.94 ± 1.41	−1.07 ± 1.21	< 0.001^b,c,d,e^
Current tobacco consumer (%)	44.1	44.7	34.7	25.7	0.001^c,e^
Muslim (Hindu as referent) religion (%)	3.6	1.8	5.5	5.1	0.24
***Wealth and possession attributes***					
Wealth index	11.00 ± 4.31	9.56 ± 4.35	12.17 ± 4.81	11.73 ± 5.10	< 0.001^a,d,e^
High-quality (*pucca*) housing (%)	10.7	8.3	20.7	18.8	0.001^b,d,e^
Land ownership (acres)	1.50 ± 1.85	1.20 ± 1.54	1.21 ± 1.86	1.07 ± 1.99	0.11
Livestock ownership (%)	51.8	46.3	34.5	25.5	< 0.001^b.c,e^
In-house tap water (%)	7.5	5.5	11.7	9.4	0.18
***Physical activity habits***					
Physical Activity (hours/day of moderate physical activity)	4.55 ± 3.66	3.98 ± 3.43	3.59 ± 3.77	3.38 ± 3.77	0.008^c^
Sedentary time (hours/day)	4.06 ± 2.50	4.42 ± 2.85	4.76 ± 2.91	4.9 ± 2.70	0.009^3^
Television time (hours/day)	1.33 ± 1.26	1.23 ± 1.24	1.68 ± 1.36	1.81 ± 1.33	< 0.001^c,d,e^
Labour occupation (%)	54.7	64.5	47.6	44.9	0.001^d,e^
***Dietary intake*** (g/1000 kcal unless otherwise specified)					
Current alcohol consumer (%)	52.3	41.7	49.7	44.2	0.10
Total energy intake (kcal/day)	2436 ± 868	2307 ± 634	2436 ± 694	2353 ± 643	0.20
Carbohydrates	179.8 ± 15.6	183.2 ± 13.3	176.3 ± 14.7	176.7 ± 12.9	< 0.001^d,e^
Protein	25.8 ± 2.1	25.3 ± 1.8	25.8 ± 1.9	25.7 ± 1.7	0.03^1^
Total fat	19.2 ± 5.5	18.3 ± 5.1	20.9 ± 5.0	21.3 ± 4.8	< 0.001^b,c,d,e^
Dietary fibre	22.4 ± 5.2	22.6 ± 5.3	21.3 ± 5.0	20.7 ± 4.6	< 0.001^c,d,e^
Dairy products	81.0 ± 73.0	75.0 ± 65.4	81.6 ± 65.3	89.2 ± 65.1	0.29
Pulses and legumes	26.4 ± 11.4	26.2 ± 12.1	28.7 ± 12.2	29.2 ± 10.7	0.005
Meat and poultry	3.8 ± 4.9	2.4 ± 2.5	3.4 ± 03.9	2.8 ± 03.9	< 0.001^a^
Fruits and vegetables	73.0 ± 46.7	68.7 ± 42.4	83.3 ± 49.0	87.2 ± 56.1	< 0.001^d,e^
Refined grains	63.6 ± 34.3	63.3 ± 31.9	71.5 ± 27.0	74.4 ± 32.7	0.001^c,e^

*P-values are for Pearson’s chi square for proportions and one-way analyses of variance (ANOVA) for means

^a^Category 1 versus 2 different to p < 0.05 with Sidak pairwise comparison; ^b^Category 1 versus 3 different to *p* < 0.05 with Sidak pairwise comparison; ^c^Category 1 versus 4 different to p < 0.05 with Sidak pairwise comparison; ^d^Category 2 versus 3 different to p < 0.05 with Sidak pairwise comparison; ^e^Category 2 versus 4 different to *p* < 0.05 with Sidak pairwise comparison; ^f^Category 3 versus 4 different to p < 0.05 with Sidak pairwise comparison

**Table 4:** Clinical, sociodemographic, and dietary characteristics by double burden diagnostic category in a sample of adults from rural South India

Characteristic	Category 1: Neither anemia nor diabetes (***n*** = 348)	Category 2: Anemia only (***n*** = 313)	Category 3: Diabetes only (***n*** = 46)	Category 4: Co-morbid anemia and diabetes (***n*** = 42)	p-value for trend*
***Descriptive characteristics***					
Age	44.6 ± 14.0	48.7 ± 15.4	48.9 ± 12.7	54.9 ± 13.4	< 0.001^a,c^
Women (%)	44.2	67.8	45.7	54.8	< 0.001^a,d^
Hypertension (%)	25.8	28.2	56.5	46.3	< 0.001^b,c,d^
Rurality Index	0.14 ± 1.77	0.092 ± 1.60	− 096 ± 1.96	−0.83 ± 1.49	< 0.001^b,c,d,e^
Current tobacco consumer (%)	41.2	37.2	37.0	38.1	0.76
Family history of diabetes (%)	7.7	7.6	36.9	28.6	< 0.00^b,c,d,e^
Muslim (Hindu as referent) religion (%)	3.4	2.2	10.9	9.5	0.006^d^
***Wealth and possession attributes***					
Wealth index	11.38 ± 4.45	10.39 ± 4.71	11.85 ± 5.14	10.50 ± 5.22	0.02
Pucca housing (%)	13.1	11.8	23.9	16.7	0.14
Land ownership (acres)	1.42 ± 1.83	1.22 ± 1.79	1.18 ± 2.04	0.68 ± 1.09	0.06
In-house tap water (%)	7.1	6.4	23.9	11.9	< 0.001^b,d^
***Physical activity habits***					
Physical Activity (hours/day of moderate physical activity)	4.47 ± 3.73	3.92 ± 3.56	2.16 ± 3.00	2.47 ± 3.44	< 0.001^b,c,d^
Sedentary time (hours/day)	4.19 ± 2.63	4.53 ± 2.78	5.38 ± 2.87	5.33 ± 2.86	0.007^c.d^
Television time (hours/day)	1.45 ± 1.31	1.49 ± 1.31	1.55 ± 1.29	1.19 ± 1.25	0.57
Labour occupation (%)	53.0	58.8	45.7	42.9	0.09
Livestock ownership (%)	48	39.6	26.1	28.6	0.003^b^
***Dietary intake*** (g/1000 kcal unless otherwise specified)					
Current alcohol consumer (%)	51.6	43.6	50	35.7	0.08
Total energy intake (kcal/day)	2451 ± 829	2349 ± 649	2324 ± 626	2153 ± 519	0.04
Carbohydrates	179.2 ± 14.8	181.0 ± 13.4	174.1 ± 18.2	178.7 ± 14.7	0.02^4^
Protein	25.8 ± 02.1	25.4 ± 01.7	25.0 ± 01.9	25.8 ± 01.6	0.06
Total fat	19.7 ± 5.3	19.4 ± 5.2	21.3 ± 5.5	20.1 ± 5.0	0.16
Dietary fibre	220.0 ± 51.6	219.6 ± 81.6	220.6 ± 108.0	217 ± 63.9	0.99
Dairy products	79.2 ± 71.4	79.6 ± 65.0	95.3 ± 58.9	89.2 ± 69.3	0.38
Pulses and legumes	27.5 ± 11.6	27.5 ± 11.9	28.3 ± 13.3	27.4 ± 09.3	0.97
Meat and poultry	3.7 ± 4.5	2.6 ± 3.1	3.7 ± 4.6	2.5 ± 3.2	0.003^a^
Fruits and vegetables	77.0 ± 48.3	76.6 ± 50.7	74.9 ± 43.3	70.4 ± 33.1	0.86
Refined grains	67.1 ± 32.3	76.6 ± 50.7	74.9 ± 43.3	70.4 ± 33.1	0.86

*P-values are for Pearson’s chi square for proportions and one-way analyses of variance (ANOVA) for means

^a^Category 1 versus 2 different to p < 0.05 with Sidak pairwise comparison; ^b^Category 1 versus 3 different to p < 0.05 with Sidak pairwise comparison; ^c^Category 1 versus 4 different to *p* < 0.05 with Sidak pairwise comparison; ^d^Category 2 versus 3 different to p < 0.05 with Sidak pairwise comparison; ^e^Category 2 versus 4 different to p < 0.05 with Sidak pairwise comparison; ^f^Category 3 versus 4 different to p < 0.05 with Sidak pairwise comparison

Several factors were associated with co-morbid anemia and overweight in age- and sex-adjusted logistic regression model (Table 5). High caste was associated with increased odds of both overweight and co-morbid anemia and overweight. Wealth index values were associated with increased odds of overweight and co-morbid anemia and overweight, indicating that wealthier individuals were at higher risk of these outcomes. Rurality index values were negatively associated with overweight and co-morbid anemia and overweight, indicating individuals from less rural households experienced a greater risk of having these conditions. Tobacco consumption (current use of *paan*^1^ or cigarettes) was negatively associated with co-morbid anemia and overweight. Finally, livestock ownership and meat intake were both negatively associated with co-morbid anemia and overweight.

**Table 5:** Factors associated with double burden categories in a multivariable logistic regression analysis in a sample of adults in rural Tamil Nadu, South India

	Anemia only OR (95% CI)	Overweight only	Co-morbid anemia and overweight
Age (continuous)	1.03 (1.02, 1.05)^a^	1.01 (1.00, 1.03)	1.01 (0.99, 1.03)
Female sex (male as referent)	3.0 (2.03, 4.46)^a^	1.30 (0.82, 2.05)	2.31 (1.39, 3.85)^a^
High caste (Brahmin)	–	3.95 (1.75, 8.93)^a^	3.17 (1.34, 7.49)^a^
Wealth index	–	1.06 (1.01, 1.13)^a^	1.05 (1.00, 1.12)^b^
Rurality index	–	0.69 (0.58, 0.81)^a^	0.69 (0.56, 0.85)^a^
Tobacco consumption	–	–	0.55 (0.32, 0.96)^b^
Livestock ownership	–	–	0.51 (0.29, 0.89)^b^
Meat and poultry intake (g/1000 kcal)	–	–	0.75 (0.61, 0.94)^b^

Only variables associated with one or more outcome level (p < 0.05) are displayed

The dependent variable is a multi-level outcome: 0 = neither anemia nor overweight (referent, not shown); 1 = anemia only; 2 = overweight only; 3 = both anemic and overweight

Definitions as follows: anemia, Hb < 130 g/dL for men, < 120 g/dL for women; overweight, ≥23 kg/m^2^

^a^p < 0.01; ^b^p < 0.05; ^c^p < 0.1; ^d^p < 0.2

Several factors were associated with co-morbid anemia and diabetes in the age- and sex-adjusted logistic regression model (Table 6). High caste was positively associated with co-morbid anemia and diabetes. Greater rurality index value was associated with lower odds of diabetes and co-occurrence of anemia and diabetes. Meanwhile, family history of diabetes was associated with much greater odds of diabetes and co-morbid anemia and diabetes.

**Table 6:** Factors associated with double burden categories in a multivariable logistic regression analysis in a sample of adults in rural Tamil Nadu, South India

	Anemia only, fully adjusted model OR (95% CI)	Diabetes only, fully adjusted model OR (95% CI)	Co-morbid anemia and diabetes, fully adjusted model OR (95% CI)
Age (continuous)	1.02 (1.01, 1.03) ^a^	1.02 (1.00, 1.04)^d^	1.08 (1.05, 1.11)^a^
Female Sex (male as referent)	2.73 (1.97, 3.79) ^a^	1.43 (0.61, 2.11)	1.04 (0.49, 2.20)
Scheduled caste or tribe (Y/N)	–	2.89 (1.21, 6.90)^b^	–
Seasonal migrant (Y/N)	0.54 (0.31, 0.94)^b^	–	–
Livestock ownership (Y/N)	0.68 (0.49, 0.94) ^b^	–	–
Rurality index	–	–	0.75 (0.57, 0.98)^b^
Family history of diabetes (Y/N)	–	4.17 (1.80, 9.62)^a^	4.86 (1.86, 12.70)^a^
Physical Activity (h/day moderate activity)	–	0.85 (0.76, 0.96)^a^	–
Body Mass Index (standardized)	–	1.87 (1.25, 2.81)^a^	2.14 (1.45, 3.14)^a^
Waist circumference (standardized)	–	1.68 (1.09, 2.57)^b^	–
Meat and poultry intake (g/1000 kcal)	0.87 (0.78, 0.98)^b^	–	–

Only variables associated with one or more outcome level (p < 0.05) are displayed

The dependent variable is a multi-level outcome: 0 = neither anemia nor diabetes (referent, not shown); 1 = anemia only; 2 = diabetes only; 3 = both anemia and diabetes

Definitions as follows: anemia, Hb < 130 g/dL for men, < 120 g/dL for women; diabetes, proof of previous diagnosis and/or CBG ≥7 mmol/L (≥126 mg/dl) and/or a 2 h post prandial CBG value ≥12.2 mmol/L (≥220 mg/dL); ^a^p < 0.01; ^b^p < 0.05; ^c^p < 0.1; ^d^p < 0.2

## Discussion

Indicators of over- and undernutrition were widespread, both at the population level and within individuals. Prevalence of most measures of over- and undernutrition in the study population were similar or higher than state-level rural averages and previous regional studies conducted in South India [7, 8, 41–44]. Underweight was more common among men, which is unusual for an Indian sample population [7, 44, 45]. Evidence suggests that anemia and underweight have been declining across India in the past decade, so timing of studies may account for differences in published data [7].

Results indicate that rural regions in South India may mirror patterns seen in urban India over the past two decades, with the burden of overweight and associated morbidities surpassing that of undernutrition [46]. The study population had similar or slightly higher prevalence of overweight and associated morbidities in comparison to previous studies in rural India and Tamil Nadu [41, 42]. As discussed elsewhere [47], this study recorded one of the highest regional burdens of diabetes in rural India at 10.8%, which is higher than state-level estimates (7.8% as measured by Anjana et al. 2011) [8] and most previous regional estimates (see Misra et al. 2011 for review of prevalence studies in rural India) [48], but was similar to a recent cross-sectional study conducted in clusters of villages in nearby Vellore, Tamil Nadu (11.2%) [43]. Prevalence of overweight (34.3% in men and 38.6% in women) was much higher than state-level rural estimates in 2006 (22.5% in men and 25.1% in women according to the National Nutritional Monitoring Board) [44], but similar to other recent regional studies in South India [41, 49]. High blood pressure (31.1% in men and 28.8% in women) was more prevalent than state-level estimates (17.6% among men and 11.5% in women as measured by IIPS) [7] and regional population studies [43, 50]. Our results corroborate recent evidence suggesting that low-resource rural regions are experiencing high rates of obesity, diabetes, hypertension, and other indicators of over-nutrition.

As yet, few studies in India have reported on the emerging double burden of malnutrition, and even fewer have investigated individual-level co-occurrence of over- and undernutrition. Alarmingly, we found that 13.1% of men and 23.1% of women had co-occurring anemia and overweight, which was considerably higher than figures reported by Jones and colleagues in 2016 (1.3% in men and 9% in women) in an urbanizing rural region of South India [21]. We also found that about half of all individuals with diabetes also had anemia. While no other studies have examined co-occurring anemia and diabetes in India, Jones and colleagues found prevalence of co-occurring anemia and metabolic syndrome (defined as three of five of abdominal obesity, high triglycerides, low HDL cholesterol, hypertension, or high blood glucose) was 2.8%, including 1.2% among men and 4.5% among women [21].

To our knowledge, this is the first cross-sectional study to assess associations between individual-level double burden of malnutrition and a wide range of demographic, socio-economic, dietary, and lifestyle risk factors in a rural region of India using multivariable logistic regression models. Several factors were associated with double burden outcomes. Our results corroborate evidence from India and other LMICs including China and Burkina Faso that co-morbid anemia and overweight or diabetes affect a larger proportion of women than men [19, 21, 51]. In addition, female sex was associated with higher odds of co-morbid anemia and overweight in multivariable models. This may be driven by Indian women being at higher independent risk of anemia, overweight, and diabetes compared to men [21, 49, 52]. It should be noted that such findings may also reflect intra-household dynamics and gender inequities that disproportionately impact women’s food intake and nutrition. For example, some studies suggest that men eat first in many Indian households, and that female children may be neglected in favour of male children [53–56]. Such inequities may exacerbate the double burden of malnutrition among women and explain the higher prevalence and co-occurrence of anemia, overweight, and diabetes compared to men.

Socio-economic status (SES) and caste in rural India are intricately linked, and several researchers concur that elevated SES and high caste are positively associated with higher risk of obesity and non-communicable diseases (NCDs) [57, 58]. In age- and sex-adjusted multivariable models, higher wealth index values were associated with greater odds of overweight and co-morbid anemia and overweight. In addition, high caste (Brahmin caste) was associated with increased odds of co-morbid anemia and diabetes, while low caste (scheduled caste or tribe status) was associated with decreased odds of co-morbid anemia and overweight. These results indicate that individuals of higher SES and higher caste were more likely to suffer from the effects of simultaneous over- and undernutrition, perhaps due to dietary and lifestyle patterns associated with wealth and caste [59]. While the effects of caste on health and disease are complex, some evidence suggests that high caste households tend to have higher standards of living, increased income, greater access to sedentary pastimes, and increased usage of vehicles, all of which may impact the risk of obesity and NCDs [60, 61]. While one might expect that higher caste and SES might reduce risk of anemia and co-morbid anemia due to improved food access [62], this does not appear to be true for this study population. Such findings correspond with previous studies that demonstrated a connection between wealth and a diet high in calories but low in micronutrients [63, 64]. Additionally, our results align with research from Jones and colleagues, who also found that their asset-based wealth index was associated with an increased odds of co-occurring anemia with overweight or metabolic syndrome [21].

The rise of NCDs in India is often attributed to urbanization. While our study region was primarily agricultural and was classified as rural by Census India definitions [65], we employed a rurality index to assess the impacts of remoteness and population density on measures of over- and undernutrition. We found strong negative associations between rurality and risk of obesity, diabetes, co-morbid anemia and obesity, and co-morbid anemia and diabetes. These findings parallel previous research in India [21] and sub-Saharan Africa [20] and may reflect urbanization-induced characteristics in the food and physical environments that promote obesity, diabetes, and other cardio-metabolic diseases [38, 66]. Such characteristics may include convenient access to shops to purchase snack foods and sweetened beverages, reduced physical activity due to proximity of amenities, and social networks and employment opportunities contributing to elevated SES [61, 67, 68]. This is an important finding, as it likely reflects the considerable variability and health implications of socio-economic, lifestyle, and dietary patterns occurring within rural regions of South India. Further, these findings underscore the importance of more nuanced approach to examining the urban-rural continuum in India, perhaps by eliminating the rural/urban dichotomy of most censuses and population health studies in favour of validated rurality or urbanicity indices or categories [69, 70].

Studies from the United States have suggested that a “westernized” diet consisting of energy-dense, but micronutrient-poor foods, may contribute to concurrent obesity and micronutrient deficiencies [67]. India is undergoing a nutrition transition characterized by a decline in the per capita consumption of traditional whole grains (e.g. small millets, barley, and buckwheat) and a diversification of food consumption [71]. In some ways, this shift mirrors prior changes in many high-income countries, including increased intake of refined sugars, saturated fats, and animal products [4, 68]. India’s nutrition transition is driven by rising incomes, economic development, urbanization, increased access to processed foods, changing food preferences, and shifting agricultural patterns, all of which are influenced by government policy and market forces [4, 59]. Of concern in rural regions is the increasing popularity of refined grains (e.g. polished white rice) which have been processed to eliminate the bran and germ, thus removing fibre, vitamins, and other compounds that may protect against micronutrient deficiencies, diabetes, and other NCDs [72]. Polished white rice consumption has increased due, in part, to national food programs such as the Public Distribution System (PDS), the Integrated Child Development Services (ICDS), and the Mid-Day Meal Scheme (MMS), all of which now fall under India’s National Food Security Act (NFSA) of 2013 and promote rice and wheat as staple sources of calories [56, 73] While these food programs have notably contributed to reducing the burdens of food insecurity and acute malnutrition in India, they have been criticized for relying heavily on staple grain distribution, thereby contributing to diets high in refined carbohydrates, low in protein, and lacking in adequate nutritional quality to prevent micronutrient deficiencies [73–75]. Indeed, some studies have suggested that by improving access and affordability of refined grains, the PDS and MMS may be exacerbating the burden of overweight and diabetes in rural India [76–79]. However, it should be noted that the infrastructure of the PDS, ICDS, and MMS represents an important opportunity to simultaneously promote calorie adequacy and improved nutrition [79, 80]. To address the double burden of over- and undernutrition among poor populations in rural India, it is necessary to leverage the reach of the NFSA and associated social welfare programs to promote the consumption of whole grains and nutrient-dense foods [74]. In some regions, targeted pilot programs have distributed whole grains (e.g. small millets) through the PDS and have seen some preliminary success [81, 82]. Such efforts should be applauded and expanded if the Government of India wishes to address the double burden of malnutrition and prevent costly future healthcare expenditures.

There is some evidence to suggest that micronutrient deficiencies may contribute to the development and exacerbation of NCDs, and conversely that NCDs may affect absorption of micronutrients, thus exacerbating micronutrient deficiency [83]. For example, micronutrients such as Vitamin C and zinc have antioxidant effects, and oxidative stress has been linked to the development and prognosis of cardiovascular disease and diabetes [84, 85]. Evidence also suggests that obesity and some NCDs further exacerbate oxidative stress, and may interact with dietary deficiencies to produce worse health outcomes [86, 87]. Similarly, some studies indicates that inflammation caused by obesity and diabetes reduces iron absorption, which may contribute to iron-deficiency anemia in individuals with these conditions [88, 89]. Such complex physiological pathways may partially explain why body mass index and waist-hip-ratio were positively associated with co-morbid anemia and diabetes in the study population. In addition, the coexistence of underweight, anemia, and diabetes appears consistent with malnutrition-related diabetes or fibrocalculous pancreatic diabetes (FCPD), for which malnutrition and micronutrient deficiencies may be etiological factors [90]. It is possible that some individuals in the study sample were misdiagnosed with type 2 diabetes when they in fact suffered from FCPD; however, considering the low prevalence of FCPD in other regions of South India (e.g. 0.019% in urban Chennai and 0.13% in rural Kerala), misclassification in this population was likely nonexistent or negligible [90–92]. Clearly, there are several potential links between nutrition intake, micronutrient deficiency and NCDs that need to be explored in further detail and may explain the high prevalence of co-morbidity in the present study.

The findings of this study are relevant to public health and clinical practice. High prevalence of co-morbid over- and undernutrition underscore the importance of public health programs, policies, and healthcare practitioners to promote education, availability, and affordability of healthy diets and lifestyle patterns that simultaneously improve dietary deficiencies and reduce burdens of NCDs. Establishing healthy food environments, simultaneous screening and health monitoring of malnutrition and cardio-metabolic health outcomes, and promoting evidence-based and culturally-sensitive behaviour change may be integral to public health approaches [20]. Our study findings suggest that screening and interventions aiming to reduce the individual-level double burden of malnutrition in India should target women living in moderately rural and urbanizing regions with a family history of metabolic disorders. Our analyses indicate that livestock ownership and meat and poultry consumption were associated with reduced odds of co-morbid overweight and anemia, suggesting that dietary interventions, and in particular improved access to nutrient-dense foods, may be beneficial to prevent or reduce this double burden pairing. Meanwhile, healthcare professionals should consider the risk of iron deficiency and anemia in all patients with obesity or cardio-metabolic disorders before recommending dietary and lifestyle changes. Our findings provide further evidence cautioning against interventions to reduce obesity through caloric restriction, as this may exacerbate nutrient deficiencies if the patient’s diet is nutritionally poor [93]. Due to the limited research on the double burden of malnutrition in India, there is a need for further observational and experimental data to determine the effectiveness of policy, public health interventions, and clinical practices in preventing and managing co-occurring over- and undernutrition.

This study had several limitations. Although we used systematic random sampling to ensure internal validity, the sample is likely not representative of the state or national rural population, and thus our findings cannot be generalized to other populations in India. Cross-sectional study designs have known limitations regarding causal interpretations of observed associations and potential confounding bias. In addition, although we mostly employed standardized and validated data collection tools, there were some notable exceptions. The asset-based wealth index was modified from the one used by the NFHS and was not validated against other measures of wealth. The rurality index was adapted from one developed for health research in the United States, but was not previously validated for use in India [39]. Although the FFQ was validated for use in rural Tamil Nadu [28], the limitations of FFQs are well-documented and include a susceptibility to social desirability bias and a tendency to overestimate food intakes [28]. Finally, due to limited access to laboratories and transportation constraints, we measured CBG, which has a wider coefficient of variation than venous plasma BG [8]. However, previous studies have shown good correlation between CBG and venous plasma estimations, and the WHO recommends CBG in low-resource settings [31].

## Conclusion

Over- and undernutrition should not be considered distinct conditions at opposite ends of the nutrition spectrum; rather, they may occur simultaneously in populations, households, and individuals. While the burden of chronic disease in India was previously socially and geographically segregated, our results suggest that as the nutrition transition progresses, obesity and associated cardiometabolic outcomes are increasingly affecting poor and rural populations. We found high prevalence of co-morbid anemia and overweight, as well as co-morbid anemia and diabetes, indicating that the double burden of malnutrition is now a severe public health concern in rural regions of South India. Women are in this rural region of South India bear a larger burden of anemia, excessive adiposity, and associated cardio-metabolic illness. Such burdens are likely further exacerbated by low literacy and education among rural women. The positive association of household wealth and decreased rurality with these conditions suggests that co-occurring over- and undernutrition will not decline with economic development and urbanization, and substantial investments in rural education and health services are likely necessary. The double burden of malnutrition in rural India is a public health crisis that must be addressed through research, healthy policy, public health education and programming, and clinical practice, particularly in the context of a rapid nutrition transition.

## Data Availability

The datasets generated and analysed during the current study are not publicly available due to research participant privacy/consent agreements. Any request for raw data will be reviewed by the corresponding author.

## Abbreviations

BMI: Body Mass Index
CBG: Capillary Blood Glucose
DBP: Double Burden Pair
FCPD: Fibrocalculous Pancreatic Diabetes
FFQ: Food Frequency Questionnaire
GPAQ: Global Physical Activity Questionnaire
ICDS: Integrated Child Development Services
IFG: Impaired Fasting Glucose
IGT: Impaired Glucose Tolerance
IIPS: International Institute of Population Sciences
LMICs: Low- and Middle-Income Countries
MMS: Mid-Day Meal Scheme
NCDs: Noncommunicable Diseases
NFHS: National Family Health Survey
NFSA: National Food Security Act
SES: Socioeconomic Status
PDS: Public Distribution System
WHO: World Health Organization

## Footnotes

1 A preparation of betel leaf, areca nut, and tobacco that is chewed for its stimulant and psychoactive properties
